# Crystal structure of 5-[bis­(4-eth­oxy­phenyl)amino]­thio­phene-2-carbaldehyde

**DOI:** 10.1107/S1600536814018984

**Published:** 2014-08-30

**Authors:** Jing-Yun Tan, Ming Kong, Jie-Ying Wu

**Affiliations:** a Department of Chemistry, Anhui University, Hefei 230039, People’s Republic of China; b Key Laboratory of Functional Inorganic Materials Chemistry, Hefei 230039, People’s Republic of China

**Keywords:** crystal structure, thio­phene-2-carbaldehyde, hydrogen bonding, supra­molecular chains

## Abstract

In the title compound, C_21_H_21_NO_3_S, the planes of the two benzene rings are nearly perpendicular to one another [dihedral angle = 84.50 (10)°] and they are oriented with respect to the plane of the thio­phene ring at dihedral angles of 59.15 (9) and 66.61 (9)°. In the crystal, mol­ecules are linked by weak C—H⋯O hydrogen bonds, forming supra­molecular chains propagating along the *b*-axis direction.

## Related literature   

For applications of thio­phene derivatives, see: Justin Thomas *et al.* (2008 ▶); Hansel *et al.* (2003 ▶); Mazzeo *et al.* (2003 ▶); Zhan *et al.* (2007 ▶); Bedworth *et al.* (1996 ▶); Raposo *et al.* (2011 ▶); Takekuma *et al.* (2005 ▶); Wurthner *et al.* (2002 ▶). For a related structure, see: Li *et al.* (2013 ▶).
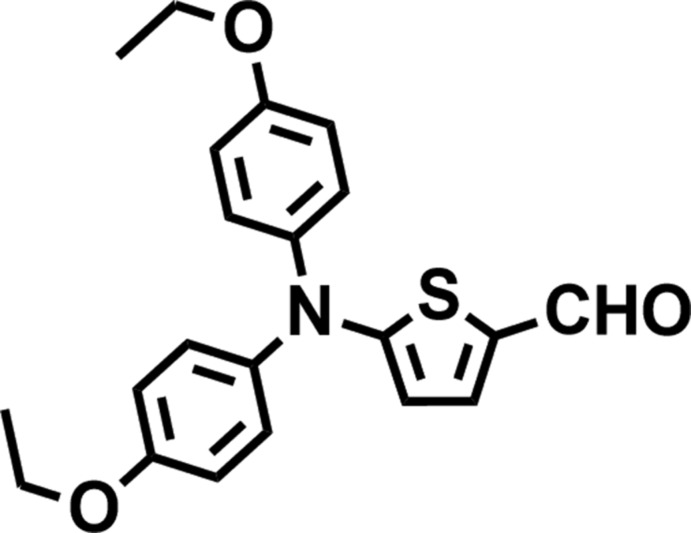


## Experimental   

### Crystal data  

C_21_H_21_NO_3_S
*M* *_r_* = 367.45
Monoclinic, 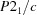

*a* = 11.101 (3) Å
*b* = 10.457 (3) Å
*c* = 17.326 (5) Å
β = 104.473 (4)°
*V* = 1947.5 (10) Å^3^
*Z* = 4
Mo *K*α radiation
μ = 0.19 mm^−1^
*T* = 296 K
0.30 × 0.20 × 0.20 mm

### Data collection  

Bruker SMART CCD area-detector diffractometer
Absorption correction: ψ scan (*SADABS*; Bruker, 2002 ▶) *T*_min_ = 0.946, *T*_max_ = 0.964
13574 measured reflections
3430 independent reflections
2596 reflections with *I* > 2σ(*I*)
*R*_int_ = 0.031

### Refinement  

*R*[*F* ^2^ > 2σ(*F* ^2^)] = 0.038
*wR*(*F* ^2^) = 0.134
*S* = 0.93
3430 reflections
237 parameters
H-atom parameters constrained
Δρ_max_ = 0.15 e Å^−3^
Δρ_min_ = −0.21 e Å^−3^

### 

Data collection: *SMART* (Bruker, 2007 ▶); cell refinement: *SAINT* (Bruker, 2007 ▶); data reduction: *SAINT*; program(s) used to solve structure: *SHELXTL* (Sheldrick, 2008 ▶); program(s) used to refine structure: *SHELXTL*; molecular graphics: *SHELXTL*; software used to prepare material for publication: *SHELXTL*.

## Supplementary Material

Crystal structure: contains datablock(s) I, Global. DOI: 10.1107/S1600536814018984/xu5814sup1.cif

Structure factors: contains datablock(s) I. DOI: 10.1107/S1600536814018984/xu5814Isup2.hkl

Supporting information file. DOI: 10.1107/S1600536814018984/xu5814Isup3.cml

**Fig. 1.**
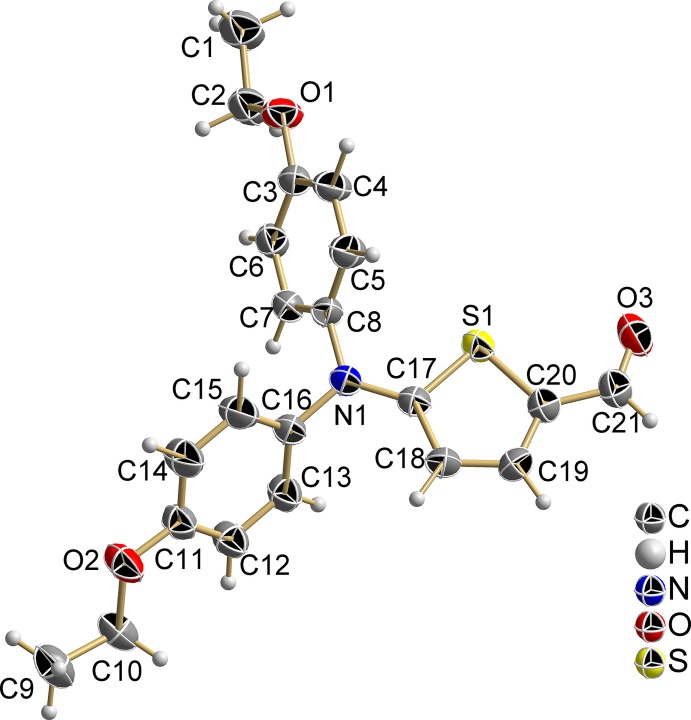
The molecular structure of the title compound, with atom labels and 50% probability displacement ellipsoids for non-H atoms

. DOI: 10.1107/S1600536814018984/xu5814fig1.tif
The mol­ecular structure of the title compound, with atom labels and 50% probability displacement ellipsoids for non-H atoms

**Fig. 2.**
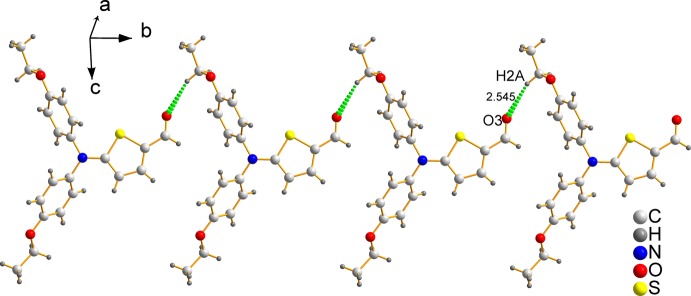
The infinite one-dimensional linear chain structure.

. DOI: 10.1107/S1600536814018984/xu5814fig2.tif
The infinite one-dimensional linear chain structure.

CCDC reference: 1016303

Additional supporting information:  crystallographic information; 3D view; checkCIF report

## Figures and Tables

**Table 1:** Hydrogen-bond geometry (Å, °)

*D*—H⋯*A*	*D*—H	H⋯*A*	*D*⋯*A*	*D*—H⋯*A*
C2—H2*A*⋯O3^i^	0.97	2.55	3.470 (3)	159

Symmetry code: (i) 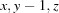.

## supplementary crystallographic information

### S1. Comment

Due to the outstanding electronic tenability and considerable chemical and environmental stability, thiophene derivatives have been widely used in solar cells (Justin Thomas *et al.*, 2008; Hansel *et al.*, 2003), organic light-emitting diodes (OLEDs) (Mazzeo *et al.*, 2003), organic field-effect transistors (OFETs) (Zhan *et al.*, 2007) and NLO devices (Bedworth *et al.*, 1996; Raposo *et al.*, 2011). Among them, the research of thiophene carboxaldehyde, which is an extremely important intermediate, is abundant (Takekuma *et al.*, 2005; Wurthner *et al.*, 2002). In this paper, a novel thiophene carboxaldehyde derivative, 5-(bis(4-ethoxyphenyl)amino)thiophene-2-carbaldehyde (Fig.1), was synthesized.

It possesses typical propeller structure, just the same with other triarylamine. The carbonyl group is coplanar with the thiophene ring, which indicates well conjugation. As shown in Fig.2, for the existence of intermolecular C2—H2A···O3 hydrogen bond, the one-dimensional linear chain structure was formed along *b* axis.

### S2. Experimental

The intermediate bis(4-ethoxyphenyl)amine was synthesized according to following procedure. Cuprous iodide (0.95 g, 5 mmol), *L*-Proline (1.15 g, 10 mmol) and anhydrous potassium carbonate (13.8 g, 100 mmol) were placed in an oven-dried 250 ml Schlenk flask. The reaction vessel was evacuated and filled with prepurified argon, a process which was repeated three times. Then refined dimethylsulfoxide (100 ml) was added with a syringe under a counterflow of argon. After that, 4-Iodophenetole (12.5 g, 50 mmol), Phenetidine (8.23 g, 60 mmol) and a particle of 18-Crown-6 (0.1981 g, 0.75 mmol) were added. The reaction was stirred at 90 degrees celsius for 24 h. Upon completion of the reaction, the mixture was cooled to room temperature. The mixture was filtered through a Buchner funnel to remove the deposition. Then diluted with water (500 ml) and stirred for one day. A kind of grey educt were obtained after separate the water by a Buchner funnel again. Purification of the residue by column chromatography on silica gel (petroleum ether/ethyl acetate = 40:1) gave bis(4-ethoxyphenyl)amine as white powder, with yield of 41.3%. *M*.p.= 89 degrees celsius. ^1^H NMR: (400 MHz, DMSO-d_6_), d(p.p.m.): 1.33 (t, 6H), 3.93 (q,4*H*), 5.61 (s, 1H), 6.80 (d, 4H), 6.96 (d, 4H).

The synthesis of the title compound. Phenanthroline (0.45 g, 2.3 mmol), cuprous iodide (0.46 g, 2.4 mmol), anhydrous potassium carbonate (5.00 g, 36 mmol) and bis(4-ethoxyphenyl)amine (3.09 g, 12 mmol) were placed in an oven-dried 250 ml Schlenk flask. The reaction vessel was evacuated and filled with prepurified argon, a process which was repeated three times. Then refined dimethylsulfoxide (120 ml) and 1.90 g 5-Bromo-2-thiophenecarbalde-hyde (10 mmol) were added with a syringe under a counterflow of argon. At last, a particle of 18-Crown-6 (0.0396 g, 0.15 mmol) and two drops of Aliquat336 (0.0200 g, 0.05 mmol) were added. The reaction was stirred at 90 degrees celsius for 48 h. Upon completion of the reaction, the mixture was cooled to room temperature. The mixture was filtered through a Buchner funnel to remove the deposition. Then diluted with water (500 ml) and stirred for one day. A kind of yellowish-brown educt were obtained after separate the water by a Buchner funnel again. Purification of the residue by column chromatography on silica gel (Petroleum/Ethyl Acetate = 20:1) gave title compound as yellowish-brown particle, with yield of 45%. m.p.= 101 degrees celsius. ^1^H NMR: (400 MHz, d-chloroform), d(p.p.m.): 1.42 (t, 6H), 4.04 (q, 4H), 6.17 (d,1*H*), 6.88 (d, 4H), 7.22 (d, 4H), 7.41 (d, 1H), 9.53 (s, 1H). ^13^C NMR(150 MHz, d_6_-acetone): d(p.p.m.): 14.81, 63.77, 109.09, 115.54, 127.17, 127.99, 128.55, 138.71, 157.39, 166.42, 180.95. IR (KBr, cm^-1^): 3058 (w), 2976 (*m*), 2931 (w), 2895 (w), 2790 (w), 1627 (*s*), 1508 (*s*), 1443 (*vs*), 1420 (*m*), 1392 (*m*), 1353 (*m*), 1244 (*s*), 1175 (*m*), 1054 (*m*), 824 (*m*).

### S3. Refinement

All hydrogen atoms were placed in geometrically idealized positions and constrained to ride on their parent atoms, with C—H = 0.93–0.97 Å, *U*_iso_(H) = 1.2 *U*_eq_(C) or 1.5*U*_eq_(C).

### Crystal data

**Table:**

C_21_H_21_NO_3_S	*F*(000) = 776
*M**_r_* = 367.45	*D*_x_ = 1.253 Mg m^−^^3^
Monoclinic, *P*2_1_/*c*	Melting point: 374 K
Hall symbol: -P 2ybc	Mo *K*α radiation, λ = 0.71073 Å
*a* = 11.101 (3) Å	Cell parameters from 3509 reflections
*b* = 10.457 (3) Å	θ = 2.3–24.1°
*c* = 17.326 (5) Å	µ = 0.19 mm^−^^1^
β = 104.473 (4)°	*T* = 296 K
*V* = 1947.5 (10) Å^3^	Block, brown
*Z* = 4	0.30 × 0.20 × 0.20 mm

### Data collection

**Table:**

Bruker SMART CCD area-detector diffractometer	3430 independent reflections
Radiation source: fine-focus sealed tube	2596 reflections with *I* > 2σ(*I*)
Graphite monochromator	*R*_int_ = 0.031
phi and ω scans	θ_max_ = 25.0°, θ_min_ = 1.9°
Absorption correction: ψ scan (*SADABS*; Bruker, 2002)	*h* = −13→13
*T*_min_ = 0.946, *T*_max_ = 0.964	*k* = −12→12
13574 measured reflections	*l* = −20→20

### Refinement

**Table:**

Refinement on *F*^2^	Primary atom site location: structure-invariant direct methods
Least-squares matrix: full	Secondary atom site location: difference Fourier map
*R*[*F*^2^ > 2σ(*F*^2^)] = 0.038	Hydrogen site location: inferred from neighbouring sites
*wR*(*F*^2^) = 0.134	H-atom parameters constrained
*S* = 0.93	*w* = 1/[σ^2^(*F*_o_^2^) + (0.1*P*)^2^] where *P* = (*F*_o_^2^ + 2*F*_c_^2^)/3
3430 reflections	(Δ/σ)_max_ < 0.001
237 parameters	Δρ_max_ = 0.15 e Å^−^^3^
0 restraints	Δρ_min_ = −0.21 e Å^−^^3^

### Special details

**Table:**

Geometry. All e.s.d.'s (except the e.s.d. in the dihedral angle between two l.s. planes) are estimated using the full covariance matrix. The cell e.s.d.'s are taken into account individually in the estimation of e.s.d.'s in distances, angles and torsion angles; correlations between e.s.d.'s in cell parameters are only used when they are defined by crystal symmetry. An approximate (isotropic) treatment of cell e.s.d.'s is used for estimating e.s.d.'s involving l.s. planes.
Refinement. Refinement of *F*^2^ against ALL reflections. The weighted *R*-factor *wR* and goodness of fit *S* are based on *F*^2^, conventional *R*-factors *R* are based on *F*, with *F* set to zero for negative *F*^2^. The threshold expression of *F*^2^ > σ(*F*^2^) is used only for calculating *R*-factors(gt) *etc*. and is not relevant to the choice of reflections for refinement. *R*-factors based on *F*^2^ are statistically about twice as large as those based on *F*, and *R*- factors based on ALL data will be even larger.

### Fractional atomic coordinates and isotropic or equivalent isotropic displacement parameters (Å^2^)

**Table:**

	*x*	*y*	*z*	*U*_iso_*/*U*_eq_	
C1	0.4645 (2)	0.2539 (2)	−0.07326 (14)	0.0795 (8)	
H1A	0.3875	0.2164	−0.1023	0.119*	
H1B	0.5294	0.1910	−0.0652	0.119*	
H1C	0.4851	0.3245	−0.1030	0.119*	
C2	0.4511 (2)	0.3002 (2)	0.00542 (12)	0.0613 (6)	
H2A	0.4353	0.2288	0.0373	0.074*	
H2B	0.5269	0.3425	0.0341	0.074*	
C3	0.32106 (17)	0.44336 (17)	0.05611 (10)	0.0441 (4)	
C4	0.22779 (19)	0.53578 (19)	0.04076 (11)	0.0528 (5)	
H4	0.1875	0.5558	−0.0116	0.063*	
C5	0.19465 (19)	0.59780 (19)	0.10249 (11)	0.0507 (5)	
H5	0.1314	0.6585	0.0919	0.061*	
C6	0.37954 (18)	0.41280 (18)	0.13421 (10)	0.0455 (5)	
H6	0.4409	0.3502	0.1451	0.055*	
C7	0.34628 (16)	0.47574 (18)	0.19585 (10)	0.0430 (4)	
H7	0.3853	0.4547	0.2483	0.052*	
C8	0.25621 (17)	0.56915 (17)	0.18073 (10)	0.0412 (4)	
C9	0.0530 (2)	0.2588 (2)	0.59350 (13)	0.0724 (7)	
H9A	−0.0314	0.2883	0.5840	0.109*	
H9B	0.0908	0.2592	0.6497	0.109*	
H9C	0.0537	0.1733	0.5733	0.109*	
C10	0.1249 (2)	0.3460 (2)	0.55176 (12)	0.0575 (6)	
H10A	0.1263	0.4323	0.5725	0.069*	
H10B	0.2100	0.3163	0.5602	0.069*	
C11	0.11284 (18)	0.4174 (2)	0.41918 (11)	0.0499 (5)	
C12	0.21873 (17)	0.49193 (19)	0.44138 (11)	0.0514 (5)	
H12	0.2638	0.4942	0.4944	0.062*	
C13	0.25766 (17)	0.56310 (19)	0.38494 (11)	0.0486 (5)	
H13	0.3301	0.6115	0.4000	0.058*	
C14	0.0478 (2)	0.4155 (2)	0.34026 (12)	0.0660 (7)	
H14	−0.0225	0.3642	0.3245	0.079*	
C15	0.08529 (19)	0.4883 (2)	0.28423 (11)	0.0586 (6)	
H15	0.0396	0.4870	0.2313	0.070*	
C16	0.19027 (17)	0.56314 (18)	0.30648 (10)	0.0427 (5)	
C17	0.25459 (15)	0.76413 (19)	0.25697 (9)	0.0397 (4)	
C18	0.22906 (18)	0.84674 (18)	0.31280 (11)	0.0460 (5)	
H18	0.1903	0.8222	0.3522	0.055*	
C19	0.26784 (18)	0.97091 (18)	0.30347 (11)	0.0499 (5)	
H19	0.2577	1.0379	0.3366	0.060*	
C20	0.32226 (17)	0.98624 (18)	0.24143 (10)	0.0462 (5)	
C21	0.3744 (2)	1.0975 (2)	0.21472 (12)	0.0575 (5)	
H21	0.3685	1.1741	0.2408	0.069*	
N1	0.22787 (14)	0.63697 (15)	0.24656 (8)	0.0450 (4)	
O1	0.34853 (14)	0.38830 (14)	−0.00900 (7)	0.0573 (4)	
O2	0.06415 (13)	0.34394 (14)	0.46905 (8)	0.0646 (4)	
O3	0.42616 (15)	1.09978 (15)	0.16028 (9)	0.0707 (5)	
S1	0.32839 (5)	0.84196 (5)	0.19335 (3)	0.0468 (2)	

### Atomic displacement parameters (Å^2^)

**Table:**

	*U*^11^	*U*^22^	*U*^33^	*U*^12^	*U*^13^	*U*^23^
C1	0.109 (2)	0.0737 (17)	0.0733 (15)	0.0184 (15)	0.0560 (15)	0.0055 (13)
C2	0.0756 (15)	0.0578 (13)	0.0612 (13)	0.0155 (12)	0.0369 (11)	0.0063 (11)
C3	0.0560 (12)	0.0403 (10)	0.0406 (9)	−0.0024 (9)	0.0208 (9)	−0.0021 (8)
C4	0.0657 (13)	0.0565 (13)	0.0369 (9)	0.0115 (10)	0.0142 (9)	0.0030 (9)
C5	0.0577 (12)	0.0504 (12)	0.0446 (10)	0.0109 (10)	0.0143 (9)	0.0024 (9)
C6	0.0505 (11)	0.0409 (11)	0.0479 (11)	0.0044 (9)	0.0176 (9)	0.0057 (8)
C7	0.0510 (11)	0.0421 (11)	0.0377 (9)	−0.0011 (9)	0.0143 (8)	0.0033 (8)
C8	0.0492 (11)	0.0393 (10)	0.0394 (9)	−0.0047 (8)	0.0192 (8)	−0.0039 (8)
C9	0.0796 (16)	0.0772 (17)	0.0695 (15)	0.0054 (13)	0.0353 (13)	0.0250 (13)
C10	0.0575 (13)	0.0674 (15)	0.0503 (12)	0.0064 (11)	0.0184 (10)	0.0179 (10)
C11	0.0493 (12)	0.0556 (13)	0.0482 (11)	−0.0079 (9)	0.0186 (9)	0.0051 (9)
C12	0.0481 (11)	0.0600 (13)	0.0435 (10)	−0.0046 (10)	0.0066 (9)	0.0085 (9)
C13	0.0437 (11)	0.0526 (12)	0.0490 (11)	−0.0091 (9)	0.0108 (9)	0.0030 (9)
C14	0.0627 (14)	0.0834 (18)	0.0522 (12)	−0.0346 (12)	0.0149 (10)	0.0007 (11)
C15	0.0595 (13)	0.0761 (15)	0.0400 (10)	−0.0205 (11)	0.0120 (9)	−0.0005 (10)
C16	0.0483 (11)	0.0436 (11)	0.0406 (9)	−0.0028 (8)	0.0192 (8)	−0.0010 (8)
C17	0.0399 (10)	0.0442 (11)	0.0361 (9)	0.0000 (8)	0.0118 (8)	0.0005 (8)
C18	0.0537 (12)	0.0484 (12)	0.0404 (10)	−0.0014 (9)	0.0201 (9)	−0.0033 (8)
C19	0.0617 (13)	0.0432 (11)	0.0459 (10)	0.0005 (9)	0.0154 (9)	−0.0083 (9)
C20	0.0526 (11)	0.0420 (11)	0.0436 (10)	0.0004 (9)	0.0113 (9)	0.0017 (8)
C21	0.0712 (14)	0.0447 (12)	0.0538 (12)	−0.0011 (10)	0.0105 (11)	0.0070 (10)
N1	0.0576 (10)	0.0412 (9)	0.0430 (9)	−0.0053 (7)	0.0254 (8)	−0.0027 (7)
O1	0.0763 (10)	0.0586 (9)	0.0431 (7)	0.0152 (8)	0.0264 (7)	−0.0011 (6)
O2	0.0617 (10)	0.0802 (11)	0.0531 (9)	−0.0194 (8)	0.0167 (7)	0.0158 (7)
O3	0.0902 (12)	0.0585 (10)	0.0688 (10)	−0.0028 (8)	0.0300 (9)	0.0201 (8)
S1	0.0575 (3)	0.0437 (3)	0.0457 (3)	−0.0001 (2)	0.0254 (2)	0.0024 (2)

### Geometric parameters (Å, º)

**Table:**

C1—C2	1.488 (3)	C10—H10B	0.9700
C1—H1A	0.9600	C11—O2	1.364 (2)
C1—H1B	0.9600	C11—C14	1.378 (3)
C1—H1C	0.9600	C11—C12	1.383 (3)
C2—O1	1.437 (2)	C12—C13	1.381 (2)
C2—H2A	0.9700	C12—H12	0.9300
C2—H2B	0.9700	C13—C16	1.378 (2)
C3—O1	1.367 (2)	C13—H13	0.9300
C3—C6	1.384 (2)	C14—C15	1.378 (3)
C3—C4	1.393 (3)	C14—H14	0.9300
C4—C5	1.377 (2)	C15—C16	1.376 (3)
C4—H4	0.9300	C15—H15	0.9300
C5—C8	1.390 (2)	C16—N1	1.437 (2)
C5—H5	0.9300	C17—N1	1.364 (3)
C6—C7	1.381 (2)	C17—C18	1.378 (2)
C6—H6	0.9300	C17—S1	1.7321 (17)
C7—C8	1.375 (3)	C18—C19	1.390 (3)
C7—H7	0.9300	C18—H18	0.9300
C8—N1	1.443 (2)	C19—C20	1.368 (2)
C9—C10	1.510 (3)	C19—H19	0.9300
C9—H9A	0.9600	C20—C21	1.427 (3)
C9—H9B	0.9600	C20—S1	1.7330 (19)
C9—H9C	0.9600	C21—O3	1.221 (2)
C10—O2	1.423 (2)	C21—H21	0.9300
C10—H10A	0.9700		
			
C2—C1—H1A	109.5	H10A—C10—H10B	108.5
C2—C1—H1B	109.5	O2—C11—C14	115.37 (17)
H1A—C1—H1B	109.5	O2—C11—C12	125.80 (17)
C2—C1—H1C	109.5	C14—C11—C12	118.83 (17)
H1A—C1—H1C	109.5	C13—C12—C11	120.11 (17)
H1B—C1—H1C	109.5	C13—C12—H12	119.9
O1—C2—C1	107.78 (18)	C11—C12—H12	119.9
O1—C2—H2A	110.2	C16—C13—C12	120.64 (17)
C1—C2—H2A	110.2	C16—C13—H13	119.7
O1—C2—H2B	110.2	C12—C13—H13	119.7
C1—C2—H2B	110.2	C15—C14—C11	121.07 (19)
H2A—C2—H2B	108.5	C15—C14—H14	119.5
O1—C3—C6	124.24 (17)	C11—C14—H14	119.5
O1—C3—C4	116.30 (16)	C16—C15—C14	120.04 (18)
C6—C3—C4	119.45 (16)	C16—C15—H15	120.0
C5—C4—C3	120.57 (17)	C14—C15—H15	120.0
C5—C4—H4	119.7	C15—C16—C13	119.28 (17)
C3—C4—H4	119.7	C15—C16—N1	118.78 (16)
C4—C5—C8	119.62 (18)	C13—C16—N1	121.92 (16)
C4—C5—H5	120.2	N1—C17—C18	128.87 (16)
C8—C5—H5	120.2	N1—C17—S1	119.71 (13)
C7—C6—C3	119.69 (17)	C18—C17—S1	111.41 (15)
C7—C6—H6	120.2	C17—C18—C19	112.33 (17)
C3—C6—H6	120.2	C17—C18—H18	123.8
C8—C7—C6	120.89 (16)	C19—C18—H18	123.8
C8—C7—H7	119.6	C20—C19—C18	114.29 (17)
C6—C7—H7	119.6	C20—C19—H19	122.9
C7—C8—C5	119.72 (16)	C18—C19—H19	122.9
C7—C8—N1	119.36 (15)	C19—C20—C21	130.17 (19)
C5—C8—N1	120.92 (17)	C19—C20—S1	110.73 (14)
C10—C9—H9A	109.5	C21—C20—S1	119.08 (15)
C10—C9—H9B	109.5	O3—C21—C20	125.0 (2)
H9A—C9—H9B	109.5	O3—C21—H21	117.5
C10—C9—H9C	109.5	C20—C21—H21	117.5
H9A—C9—H9C	109.5	C17—N1—C16	121.39 (14)
H9B—C9—H9C	109.5	C17—N1—C8	120.02 (14)
O2—C10—C9	107.39 (18)	C16—N1—C8	117.85 (15)
O2—C10—H10A	110.2	C3—O1—C2	117.19 (15)
C9—C10—H10A	110.2	C11—O2—C10	117.79 (15)
O2—C10—H10B	110.2	C17—S1—C20	91.23 (9)
C9—C10—H10B	110.2		
			
O1—C3—C4—C5	−179.17 (18)	C19—C20—C21—O3	176.6 (2)
C6—C3—C4—C5	0.8 (3)	S1—C20—C21—O3	−1.2 (3)
C3—C4—C5—C8	1.0 (3)	C18—C17—N1—C16	−13.9 (3)
O1—C3—C6—C7	178.88 (17)	S1—C17—N1—C16	167.25 (13)
C4—C3—C6—C7	−1.1 (3)	C18—C17—N1—C8	176.16 (18)
C3—C6—C7—C8	−0.4 (3)	S1—C17—N1—C8	−2.7 (2)
C6—C7—C8—C5	2.2 (3)	C15—C16—N1—C17	130.0 (2)
C6—C7—C8—N1	−177.18 (16)	C13—C16—N1—C17	−51.4 (3)
C4—C5—C8—C7	−2.5 (3)	C15—C16—N1—C8	−59.9 (2)
C4—C5—C8—N1	176.91 (17)	C13—C16—N1—C8	118.7 (2)
O2—C11—C12—C13	−179.35 (19)	C7—C8—N1—C17	113.3 (2)
C14—C11—C12—C13	0.1 (3)	C5—C8—N1—C17	−66.1 (2)
C11—C12—C13—C16	1.5 (3)	C7—C8—N1—C16	−57.0 (2)
O2—C11—C14—C15	178.1 (2)	C5—C8—N1—C16	123.60 (19)
C12—C11—C14—C15	−1.4 (4)	C6—C3—O1—C2	−4.8 (3)
C11—C14—C15—C16	1.0 (4)	C4—C3—O1—C2	175.18 (17)
C14—C15—C16—C13	0.7 (3)	C1—C2—O1—C3	−179.38 (18)
C14—C15—C16—N1	179.3 (2)	C14—C11—O2—C10	−177.71 (19)
C12—C13—C16—C15	−1.9 (3)	C12—C11—O2—C10	1.8 (3)
C12—C13—C16—N1	179.50 (18)	C9—C10—O2—C11	179.51 (18)
N1—C17—C18—C19	−178.39 (18)	N1—C17—S1—C20	178.13 (15)
S1—C17—C18—C19	0.5 (2)	C18—C17—S1—C20	−0.88 (14)
C17—C18—C19—C20	0.3 (3)	C19—C20—S1—C17	1.03 (15)
C18—C19—C20—C21	−178.9 (2)	C21—C20—S1—C17	179.24 (16)
C18—C19—C20—S1	−1.0 (2)		

### Hydrogen-bond geometry (Å, º)

**Table:**

*D*—H···*A*	*D*—H	H···*A*	*D*···*A*	*D*—H···*A*
C2—H2*A*···O3^i^	0.97	2.55	3.470 (3)	159

Symmetry code: (i) *x*, *y*−1, *z*.
