# Supplementary figures and images for: Crystal structure of 5-[bis­(4-eth­oxy­phenyl)amino]­thio­phene-2-carbaldehyde
Source: Acta Crystallogr Sect E Struct Rep Online. 2014 Aug 30;70(Pt 9):o1075–6. doi: 10.1107/S1600536814018984 (PMC4186151; doi:10.1107/S1600536814018984)

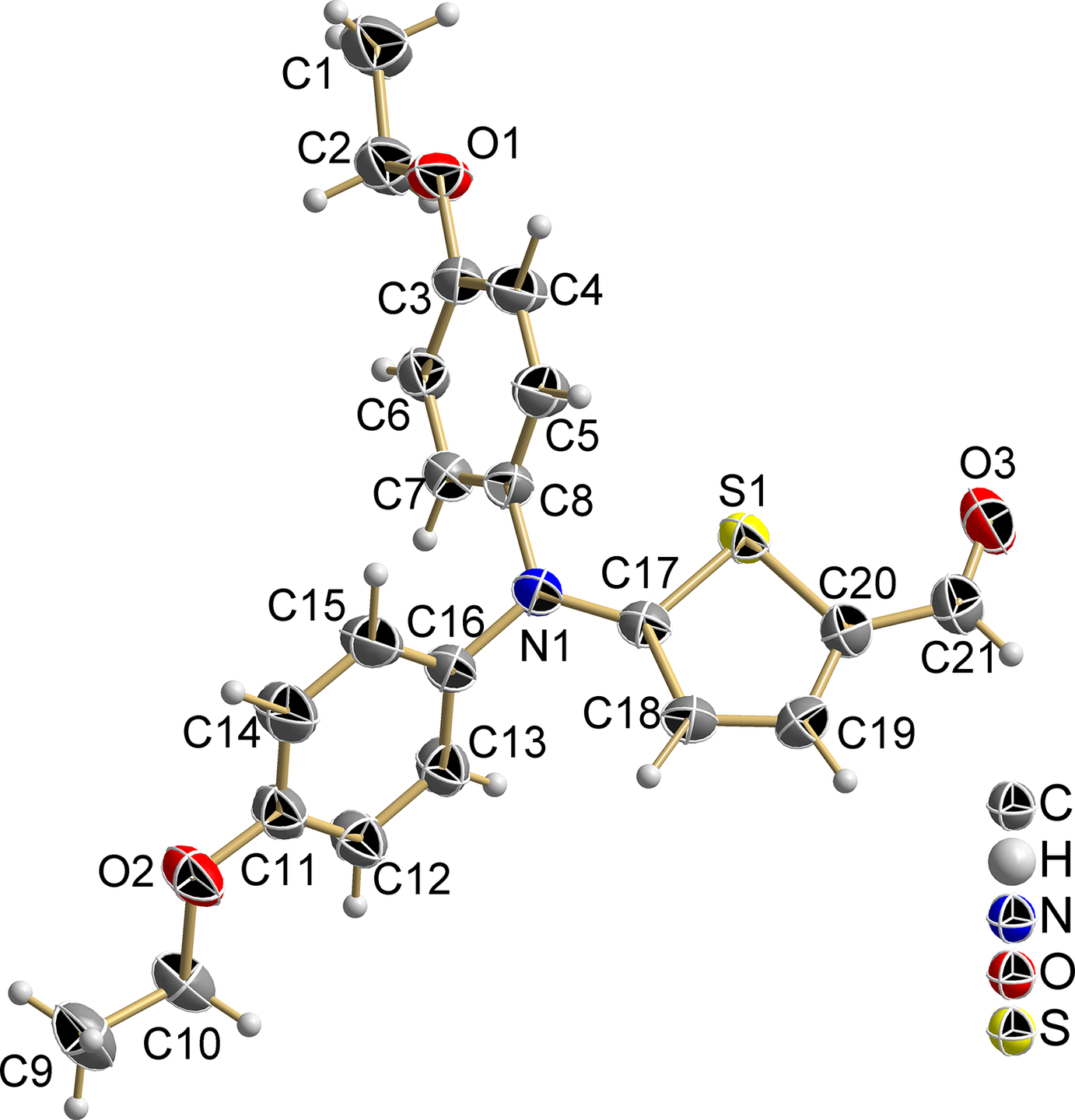

Supplement: Supplementary file 4 [file e-70-o1075-fig1.tif]

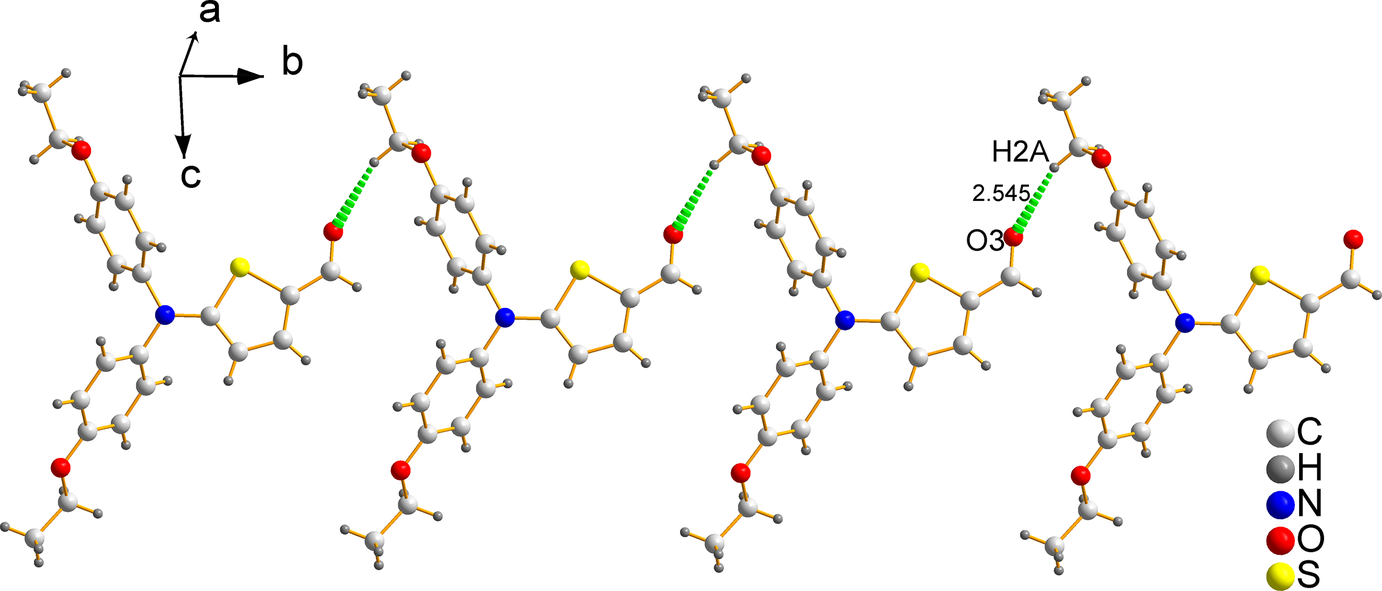

Supplement: Supplementary file 5 [file e-70-o1075-fig2.tif]
